# The role of the doctor in precision medicine

**DOI:** 10.31744/einstein_journal/2019ED4979

**Published:** 2019-06-17

**Authors:** Tatiana Ferreira de Almeida

**Affiliations:** 1 Hospital Israelita Albert Einstein, São Paulo, SP, Brazil.

Every day doctors face issues related with identical diagnoses and treatments that generate different outcomes. Being able to individualize care is an old desire of medicine. In this context, precision medicine takes into account individual genetic variability, environmental factors and lifestyle of each individual to find the best therapeutic and preventive approaches for specific groups.^(1)^ Approximately 15 years ago, the discussion about precision medicine took a new direction due to the new computational technologies and advances in molecular biology that allowed a massive growth in the amount of data.^(2)^

In general, individuals seek health care when they perceive that their usual state of health is compromised. Physician gather information and, based on clinical findings and their personal technical knowledge, make decisions about best treatment and preventive measures for the patient. In a setting including precision medicine, patient’s clinical information is collected using electronic medical record since birth, social networks indicate their habits and lifestyle; their electronic devices monitoring vital signs and moves at every second; whole genome brings 2 million different variants, besides the possibility of broaden the application of omics with transcriptome, metabolome and microbiome. All these information are processed using mathematical algorithms and they are used to establish standards and determine treatments, and preventive measures. In the same way that the algorithm can identify cats and dogs pictures, such mathematical algorithms can also identify groups of newborns at risk for hypertension, diabetes, autism.^(3)^

In this new and personalized clinical scenario, the patient seeks a doctor in his/her usual state of health and his/her clinical information is processed computationally. Medical and hospital centers will move to a secondary position as healthcare facilities, while technology companies will have the responsibility to manage individual’s data and mathematical health models. Such data, based on individuals’ personal clinical information and past events, are extremely effective to help to predict expected outcomes for individuals achieve an healthy life, without the need of pathophysiological explanation.^(4)^

In the current context, precision medicine should be applied with caution, as a way to reduce the uncertainties of the patient’s clinical management, especially issues related with treatment of tumors and pharmacogenomics. Clinical studies to prove the efficacy of genomic findings and predictive models need to be adjusted to the new reality. With the increase in the amount of data available and the reduction of groups, the current statistics and the forms how to assess clinical trials need to be adapted.^(5)^

Physicians are expected not only to define how to manage treatment of a disease, but also how to maintain and improve individuals’ health care mainly by considering patients’ information from predictive models, which can be black boxes, and also new clinical trial models.
